# 3D Cytocompatible Composites of PCL/Magnetite

**DOI:** 10.3390/ma12233843

**Published:** 2019-11-21

**Authors:** Esperanza Díaz, María Blanca Valle, Sylvie Ribeiro, Senentxu Lanceros-Mendez, José Manuel Barandiarán

**Affiliations:** 1 Escuela de Ingeniería de Bilbao, Departamento de Ingeniería Minera, Metalúrgica y Ciencia de Materiales, Universidad del País Vasco (UPV/EHU), 48920 Portugalete, Spain; 2 BCMaterials, Basque Centre for Materials, Applications and Nanostructures, UPV/EHU Science Park, 48940 Leioa, Spain; senetxu.lanceros@ehu.es (S.L.-M.); jm.barandiaran@ehu.es (J.M.B.); 3 Facultad de Ciencia y Tecnología, Departamento Electricidad y Electrónica, Universidad del País Vasco (UPV/EHU), 48940 Leioa, Spain; mariablanca.valle@ehu.es; 4 Centro de Física, Universidade do Minho, 4710-057 Braga, Portugal; sylvie.ribeiro@ehu.es; 5 Centre of Molecular and Environmental Biology (CBMA), Universidade do Minho, Campus de Gualtar, 4710-057 Braga, Portugal; 6 IKERBASQUE, Basque Foundation for Science, 48013 Bilbao, Spain

**Keywords:** PCL, magnetite, scaffolds, magnetism, cytotoxicity, in vitro degradation

## Abstract

A study of Magnetite (Fe_3_O_4_) as a suitable matrix for the improved adhesion and proliferation of MC3T3-E1 pre-osteoblast cells in bone regeneration is presented. Biodegradable and magnetic polycaprolactone (PCL)/magnetite (Fe_3_O_4_) scaffolds, which were fabricated by Thermally Induced Phase Separation, are likewise analyzed. Various techniques are used to investigate in vitro degradation at 37 °C, over 104 weeks, in a phosphate buffered saline (PBS) solution. Magnetic measurements that were performed at physiological temperature (310 K) indicated that degradation neither modified the nature nor the distribution of the magnetite nanoparticles. The coercive field strength of the porous matrices demonstrated ferromagnetic behavior and the probable presence of particle interactions. The added nanoparticles facilitated the absorption of PBS, with no considerable increase in matrix degradation rates, as shown by the Gel Permeation Chromatography (GPC) results for Mw, Mn, and I. There was no collapse of the scaffold structures that maintained their structural integrity. Their suitability for bone regeneration was also supported by the absence of matrix cytotoxicity in assays, even after additions of up to 20% magnetite.

## 1. Introduction 

Nanomaterials have attracted increasing scientific interest, due to their numerous biomedical applications. Among such materials, Iron-Oxide NanoParticles (IONPs) awaken special interest, because of their magnetic properties, biocompatibility, and nanometric sizes that can interact at the cellular, subcellular, and even molecular levels [1,2]. As iron is a metal that is found in haemoglobin and myoglobin in the form of ferritin, the particles are non-toxic, although particle aggregation can be dangerous [3,4,5]. Mainly used in biomedicine, IONPs have FDA approval for use in cellular therapy, drug delivery, contrast agents, tumor hyperthermia, and tissue repair. At particle sizes of less than 30 nm, their diameters range between 5 and 100 nm. Each particle produces a nanoscale magnetic field and acts as a single magnetic domain, showing superparamagnetic behavior [2,6,7].

Biodegradable and biocompatible polymers, such as polycaprolactone (PCL), are widely used in biomedical regenerative treatments of hard tissue [8,9,10], where prolonged degradation times require lengthy curing phases [11,12,13,14,15]. PCL scaffolds with lower molecular weights and faster degradation rates are also used in the regenerative treatment of soft tissues, such as cartilage and muscle [12,14]. 

Therefore, scaffolds have become a key material in tissue engineering, in which various biological agents interact for their correct development [3,6]. The manufacture of scaffolds with magnetic fields that attract these biological agents has implied a significant advance. A similar concept was previously used for tumor hyperthermia and the administration of pharmaceuticals [7,14,15,16]. Therefore, magnetic nanoparticles and scaffolds are promising alternatives for bone regeneration. The magnetic fields of the scaffolds function by attracting and absorbing stem cells, in vivo growth factors, and other bioactive agents tied to magnetic particles, favoring bone regeneration and repair [17,18,19,20,21,22,23]. 

There are two methods for the manufacture of magnetic scaffolds: one consists of submerging a scaffold in water-based ferrofluids that contain IONPs through capillary adsorption, as described by other authors [6,19]. The other method involves the incorporation of IONPs during scaffold preparation, a method that requires effective ultrasonic nanoparticle dispersion, to prevent agglomeration [20,21,22,23,24,25,26,27].

A simple and efficient method of incorporating IONP in a scaffold that is manufactured by lyophilization will be presented in this study, so the scaffold magnetization process will not affect the external structure of the scaffold. An in vitro degradation study of the scaffolds manufactured with this method will also be performed. In addition, the utility of these magnetic scaffolds will be demonstrated in a cytotoxicity evaluation of samples with the MC3T3-E1 pre-osteoblast cell line for bone regeneration.

## 2. Experimental

### 2.1. Materials

PCL polymer, which was supplied by Purac Biomaterials Purasorb PCL 12 (Gorinchem, Amsterdam, the Netherlands), was purified by dissolution in 1,4-dioxane (Panreac p.a. Barcelona, Spain). 1,4-Dioxane was used as a solvent. The values of Mw = 134,418, Mn = 92,103, and polydispersity Mw/Mn = 1.459 were performed while using gel permeation chromatography (GPC, Perkin Elmer 200, Bridgeport, CT, USA) in tetrahydrofuran (THF). The PBS (phosphate buffer solution) used in the in vitro degradation was supplied by Fluka Analytical (Sigma-Aldrich, Darmstadt, Hessen, Germany) at a pH of 7.2. The magnetite nanoparticles were supplied by Sigma-Aldrich), CAS Number 1317-61-9, the particle size ≈ 50 nm. 

### 2.2. Fabrication of Porous Matrices

Pure PCL and PCL/Fe_3_O_4_ composite scaffolds were made by Thermally-Induced Phase Separation (TIPS) and then by a freeze-drying technique. In brief, a solution of PLLA in 1,4-Dioxane (2.5% (w/v)). Subsequently, magnetite particles (in solid state) were homogeneously blended, in proportions of 5 and 10% of total polymer mass, by ultrasonic stirring. The solutions were frozen and freeze-dried (LyoQuest of Telstar, Barcelona, Spain) for seven days for the complete elimination of the 1,4 dioxane. 

### 2.3. In vitro Degradation

For the degradation test, the porous matrices were cut into 0.5 cm^2^ rectangular section, weighed, immersed in tubes with 15 mL of PBS, and then incubated in a thermostated oven at 37 °C. The pieces were recovered after 5, 8, 10, 16, 20, 25, and 28 weeks and then wiped. A pH meter PCE228 (PCE Instruments, Palm Beach, FL, USA) was used to determine the pH changes in the PBS. 

Water absorption (W_a_) and mass loss (W_L_) were respectively calculated by the following equations:

(1) $$W_{a}\%\;=\;\frac{\mathrm{Ww}-\mathrm{Wr}\;}{\mathrm{Wr}}\;\times \;100\%$$

(2) $$W_{L}\%\;=\;\frac{\mathrm{Wo}-\mathrm{Wr}\;}{\mathrm{Wo}}\;\times 100\%$$

where, W_0_ is the original weight, W_r_ is the residual weight after degradation and when completely dried, and W_w_ is the weight of the sample without surface water. 

### 2.4. Magnetic Analysis

A Vibrating Sample Magnetometer (VSM) that was developed at the University of the Basque Country was used for magnetic measurements at physiological temperature (310 K). Pure nickel was used to calibrate the VSM. The magnetic field range was ±1.8 Tesla (18 kG) and the resolution ±20 µTesla (0.2 G).

### 2.5. SEM Analysis

The morphology of the porous matrices was examined while using scanning electron microscopy (SEM) (HITACHI S-4800, Tokyo, Japan). Before the analysis, a JEL Ion Sputter JFC-1100 (Amiron Machinery, Oxnard, CA, USA) at 1200 V and 5 mA was used to coat with a cape of gold.

### 2.6. Differential Scanning Calorimetry (DSC)

DSC assays were made with a Q200 TA Instruments. A nitrogen purge gas was used to avoid any oxidation of the scaffolds during the experiments. These were repeated three times. In the first scan, the scaffolds were heated from −20 to 250 °C at a rate of 10 °C·min^−1^ to record the melting temperature, Tm, and the melting heat, ∆H_melt_. Subsequently, cooled at 10 °C·min^−1^ to obtain the glass transition, T_g_, and then finally heated at 10 °C/min. Crystallinity, Xc, was calculated with the following equation:

X_c_ (%) = (∆H_melt_ − ∆H_crystallization_)/∆H_100%_ × 100% (3)

∆H_melt_ (J·g^−1^ of the crystalline polymer) is the enthalpy of fusion of the sample and ∆H_100%_ is the enthalpy of fusion of a 100% crystalline polymer, which for PCL was 139.5 J·g^−1^ [24]. The crystallizable fraction (CF%) of the samples was calculated with the following equation:

CF% = (∆H_c_/∆H_m_) × 100% (4)

### 2.7. Fourier-Transform Infrared (FTIR) Spectroscopy

A Thermonicolet Avatar 370 Fourier-transform infrared (FTIR, Thermo Electron Corporation, Waltham, MA, USA) spectrophotometer that was equipped with an attenuated total reflectance attachment with ZnSe crystal was used to make the infrared spectra.

### 2.8. Cytotoxicity Assay

Membrane sterilization: For the in vitro assays, the membranes were cut with 0.1mg·mL^−1^ following an adaptation of the ISO 10993-5:2009 (third edition) standard test method (indirect cytotoxicity evaluation). The samples were sterilized by UV (1 h each side). After that, each sample was washed five times (5 min each wash) in phosphate buffered saline (PBS) solution.

Cytotoxicity process: The samples were immersed in a 24-well tissue culture polystyrene plate with DMEM obtaining like this a conditioned medium. This medium contains 1 g·L^−1^ glucose (Gibco) that was supplemented with 10% FBS (Biochrom) and 1% P/S (Biochrom) (basal medium). The plate was incubated for 24 h at 37 °C, in a 95% humidified atmosphere containing 5% CO_2_. In parallel, the MC3T3-E1 cells, Sigma-Aldrich (Darmstadt, Hessen, Germany) (passage number: 36, Riken bank) were seeded in the 96-well tissue culture polystyrene plate at a density of 3 × 10^4^ cells·mL^−1^ and then incubated for cell plate attachment (24 h). 

Thereafter, the culture medium from the 96-well tissue culture polystyrene plate was removed and 100 µL of conditioned medium was added to the wells incubating for 24 and 72 h. 3-(4,5-dimethylthiazol-2-yl)-2,5-diphenyltetrazolium bromide (MTT) assay was used to quantify the viable cells. In this method, it was used a positive control (20% solution of dimethylsulfoxide (DMSO, Sigma Aldrich)) and a negative control (cell culture fresh basal medium) to compare with the values that were obtained for the samples in study.

Likewise, the conditioned medium was removed from each well at the end of each cycle (24 and 72 h) and the MTT solution (5 mg·mL^−1^ in PBS dissolved in DMEM in proportion of 10%) was added to the cells. The incubation was done for 2 h in the dark at 37 °C. After the intended contact time, it was added 100 µL of DMSO/well to dissolve the obtained MTT crystals after removing the medium. In the final, microplate reader at 570 nm was used to measure optical density

All the quantitative results were obtained from four replicates samples and controls and they were analyzed as the average of viability ± standard deviation (SD).

## 3. Results and Discussion

In this section, the results are presented of the in vitro degradation of the magnetic scaffolds that were manufactured with PLC incorporating magnetized particles of nFe_3_O_4_.

### 3.1. Magnetism

**Figure 1:**
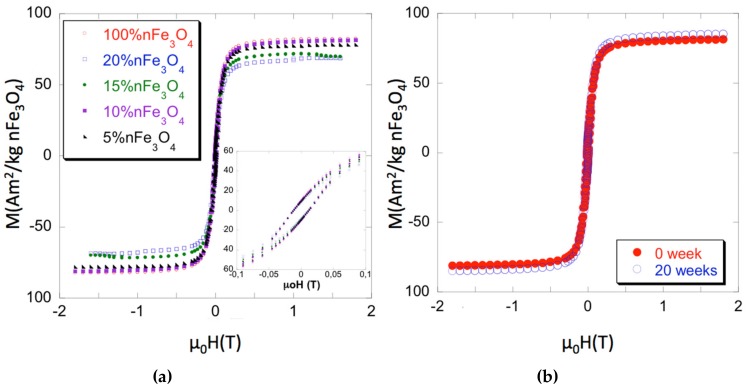
(**a**) Magnetization curves of PCL-nFe_3_O_4_. (**b**) Magnetization curves of PCL-10%nFe_3_O_4_ before and after 20 weeks degradation.

The hysteresis loops of both the Fe_3_O_4_ nanoparticles and of the PCL/nFe_3_O_4_ scaffolds are shown to be normalized to the nFe_3_O_4_ content in Figure 1a for selected measurements. Magnetic parameters that cover magnetization measured at 1.5 T (close to saturation), the coercive field (µ_0_H_c_), and the recalculated content of nanoparticles are summarized in Table 1, as the average of several measurements for each composition (typically three or four samples each). Errors have been estimated as a combination from the standard deviation of the measurements and the absolute accuracy of the instruments and they are always around 2% of the averaged value. As can be seen, the coercivity of the isolated particles and scaffolds is quite similar. Pure magnetite coercivity ≈ 12 mT (≈ 120 G) is high enough to discount classic superparamagnetic behavior. Therefore, the nanoparticle magnetic properties might be due to strong inter-particular interaction [27] or to the true ferromagnetic state, while considering that their sizes (diameters over 30 nm) are within the single domain region.

Strong interaction within the scaffolds appears to be a less likely hypothesis, as the different Fe_3_O_4_ concentrations caused no changes of coercivity. Particulate interactions can be short or long range: i.e., boundary exchanges or dipolar interactions, respectively. The latter can favor agglomerations, which leads to increased coercivity, by promoting exchange coupling within the agglomerates. Nevertheless, agglomerates are not seen in the microscopy studies, which suggest the good particulate dispersion at all concentrations, with low dispersion of inter-particle distances for each sample. Figure 1b shows the effect of 20 weeks degradation on the scaffolds PCL-10%nFe_3_O_4_. Both curves, before and after, are superimposed, which indicated that polymer degradation neither affected the characteristics nor the distribution of the magnetic nanoparticles.

### 3.2. FTIR

**Figure 2:**
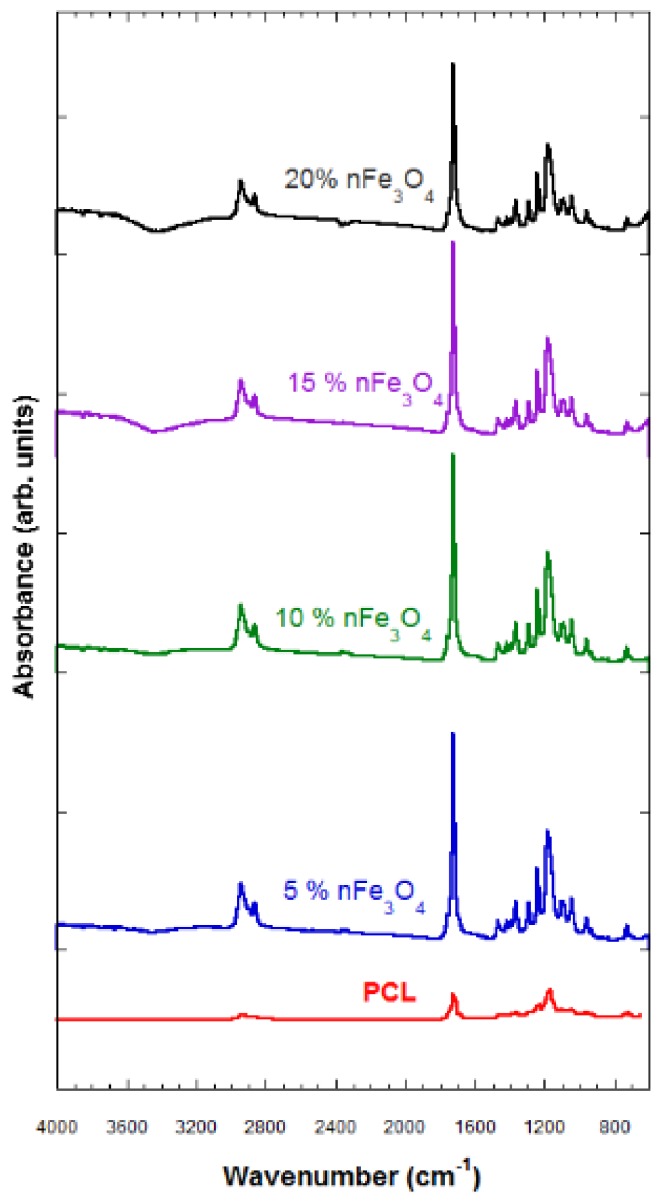
Fourier-Transform Infrared (FTIR) spectra of PCL-n Fe_3_O_4._

The FTIR technique characterizes the samples that were subjected to degradation and those that have not been degraded. The characterization through FTIR of the porous substrates composed of PCL-nFe_3_O_4_ (see Figure 2) indicated no interaction between both materials, so the magnetic nanoparticles must therefore be scattered throughout the scaffold structure.

The typical vibration bands of C=O and the stretching vibration bands of C–O to 1725 cm^−1^ and to 1293 cm^−1^, respectively, as well as the symmetric and asymmetric modes of CH_2_, at 2923 cm^−1^ and at 2940 cm^−1^, respectively, refer to the polymer (PCL).

A band of H_2_O located above 3420 cm^−1^ was observed that grew in strength when increasing the amount of magnetite; a characteristic vibration band of the Fe–O bond, hardly perceived in the compositions of 5 and 10% nFe_3_O_4_, became clearer in the PCL-nFe_3_O_4_ scaffolds containing a higher magnetite content; see Figure 2.

The hydrophilic nature of the system was reflected by the appearance of broad peaks revealing the presence of hydroxyl groups around 3420 cm^−1^ (Figure 2) for all compositions. These bands indicated the presence of water in the system, despite the lyophilization process.

**Figure 3:**
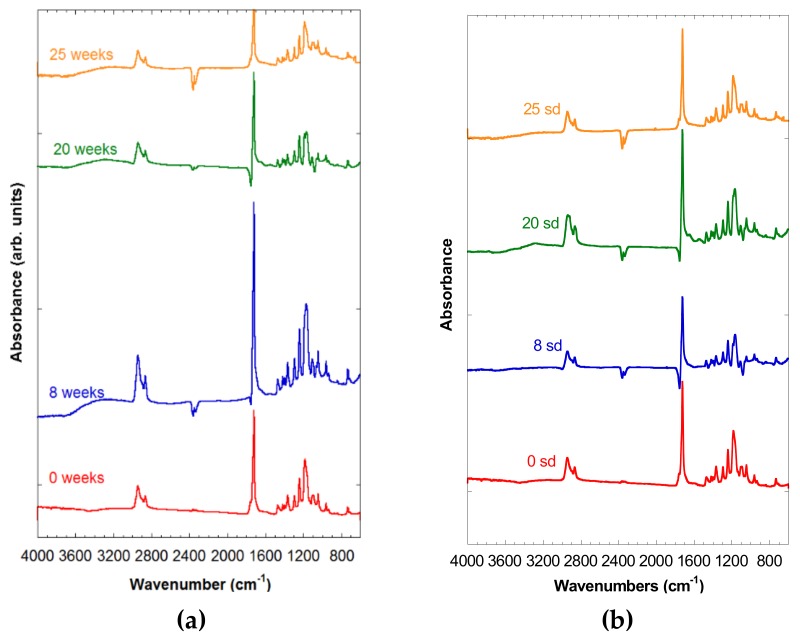
(**a**) Spectra of PCL-5% nFe_3_O_4_ after various degradation times. (**b**) FTIR spectra of PCL-10% nFe_3_O_4_ after various degradation times.

In Figure 3a,b, the spectra of both PCL-5% nFe_3_O_4_ and PCL-10% nFe_3_O_4_ were observed after 0, 8, 20, and 25 weeks of degradation, in which no appreciable signs of degradation were noted, due to the existence of the broad band corresponding to the hydroxyl groups at around 3420 cm^−1^, which might hide the appearance of a band between 2800 and 3600 cm^−1^ attributable to a stretching vibration of the OH bands of the COOH and OH groups. Neither can an increase in the intensity be observed in the region of 1100–1050 cm^−1^. Both of the changes would be due to the scission of the polymeric chains through the ester band, producing a carboxylic group and an alcohol group, as a consequence of the degradation of the PCL.

### 3.3. DSC

Table 2 shows the values of the characteristic glass-transition temperatures, T_g_, and fusion temperature (T_m_), as well as the fusion enthalpies (∆H_m_), and crystallization (∆H_c_), and the degree of crystallinity. The T_g_ temperature of the PCL-nFe_3_O_4_-porous scaffolds, −59 °C, was close to the values cited by other authors for PCL (~−60 °C).

Even though PCL is a hydrophobic polymer, the presence of magnetite nanoparticles that were embedded in the polymeric matrix make the surface hydrophilic rather than hydrophobic, improving its biocompatibility through intramolecular bridging interactions, and hydrogen bonds. However, the addition of nanoparticles modified the polymer, reducing the degree of crystallinity (X_c_%) of the PCL, which passed from a value of 77% in the scaffolds of pure polymer, to 55% in PCL-20%nFe_3_O_4_, indicating that the magnetite nanoparticles played no role as nucleation agents [28].

The T_c_ (crystallization temperature) notably increased with the addition of the nanoparticles, which entailed a much slower crystallization process of the PCL-nFe_3_O_4_ scaffolds, thereby confirming that the nanoparticles were not acting as nucleating agents. ∆H_m_ clearly diminished with the increased concentration of nanoparticles, from 107 J·g^−1^ to 81 J·g^−1^, in the pure PCL and in the PCL with a content of 20% nFe_3_O_4_, respectively.

The samples with a higher content of nanoparticles presented lower crystallinity and fusion enthalpies, due to the increased rigidity of the polymeric chains [28,29], which was clearly evident from both the crystallinity percentages and the T_g_.

During the degradation process, the T_g_ fell to temperatures lower than −59 °C in week 0 to −63 °C in week 104 for both series of compound materials. Other authors have observed this same diminishment of T_g_ in relation to the in-vitro degradation [30].

The degree of crystallinity of these samples hardly varied over the time of immersion. In addition, a stretching might be seen in the fusion temperature interval for week 25 in both compositions.

**Figure 4:**
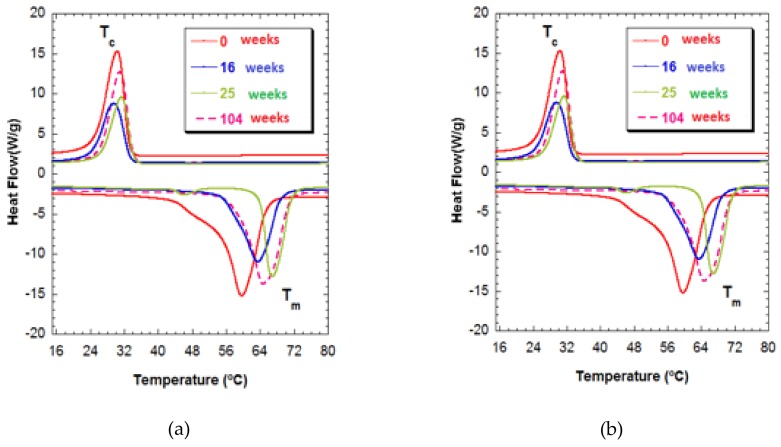
(**a**) Thermograms of PCL-5%nFe_3_O_4_ after various degradation times. (**b**) Thermograms of PCL-10%nFe_3_O_4_ after various degradation times_._

In Figure 4, T_m1_ can be seen to gain a much higher temperature of 65 °C for both compositions for week 104 of degradation and, at the same time, has a broad endothermic peak. This behavior is indicative of crystallites of different sizes, a form of degradation in which polymeric chains of different lengths are produced that are characterized by their different fusion temperatures. The shortening of the polymeric chains was unrelated to increased crystallinity. In any case, the presence of polymeric chains of different lengths makes it clear that heterogeneous degradation took place at this stage [31].

### 3.4. SEM

**Figure 5:**
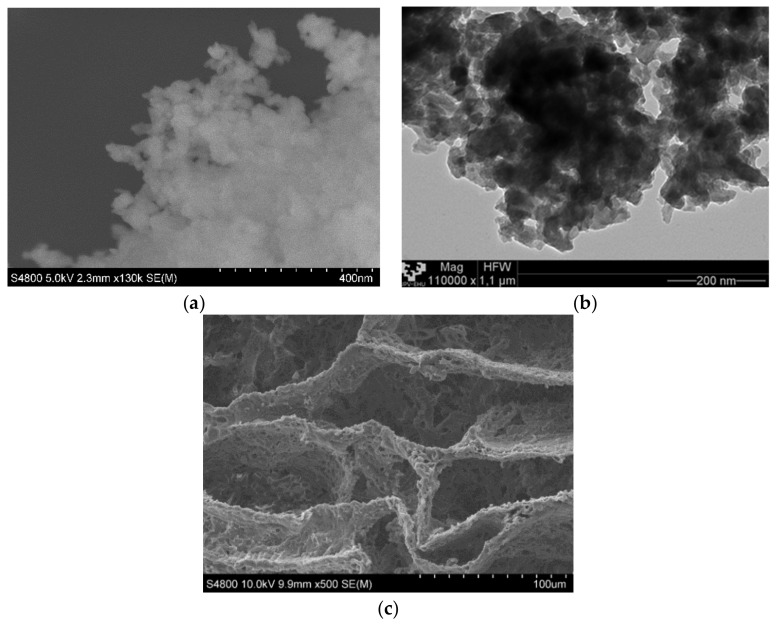
(**a**) Scanning electron microscopy (SEM) micrograph of magnetite nanoparticles. (**b**) TEM micrograph of magnetite nanoparticles. (**c**) SEM micrograph of poly(ε-caprolactone) (PCL) scaffold.

In Figure 5, we can observe the magnetite nanoparticles and the PCL scaffold both without mixing. Magnetite nanoparticles were analyzed while using TEM and SEM techniques Figure 1a,b, they are agglomerated and it was very difficult to identify single nanoparticles of ~50 nm, as indicated by the provide.

**Figure 6:**
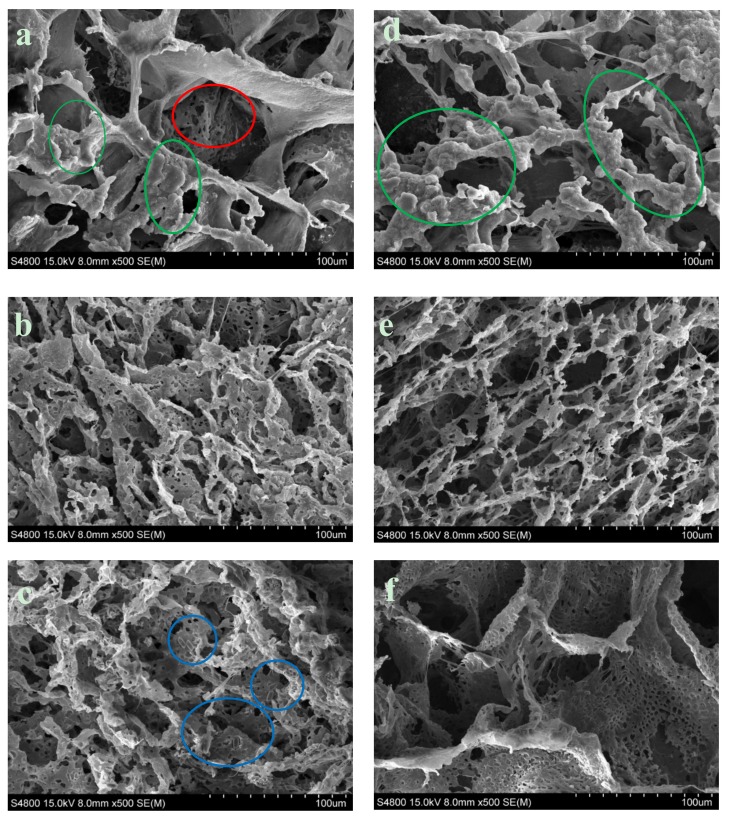
SEM observation of Surface morphology of PCL ×500. (**a**) PCL-5% nFe_3_O_4_. (**b**) PCL-5% nFe_3_O_4_ after in vitro degradation for 8 weeks. (**c**) PCL-5% nFe_3_O_4_ after in vitro degradation for 25 weeks. (**d**) PCL-10% nFe_3_O_4_. (**e**) PCL-10 % nFe_3_O_4_ after in vitro degradation for 16 weeks. (**f**) PCL-10 % nFe_3_O_4_ after in vitro degradation for 25 weeks.

The matrix manufacturing technique (TIPs) with low percentages of polymer and a volume of solvent created highly porous scaffolds with random pore structures, as may be seen in Figure 5c and Figure 6. A typical nucleation structure in all of the scaffolds, due to an excessively rapid cooling speed, was obtained, as the solid-liquid phase separation process to take place [32].

We can also see in Figure 6 that the increased content of nFe_3_O_4_ in the composition of the scaffold produced larger-sized pores and thinner walls, acquiring a more scattered appearance and more irregular pore distribution.

Measuring the exact sizes of the pores was not simple, due to the anisotropy of the porosity. In general, the macropores appeared to have average diameters of ~100 µm for the composition with 5% magnetite and ~130 µm for the composition with 10%. The diameter of the macropores fluctuated between 25–210 µm and the micropore diameter was between 2–10 µm, for the composition with 5% magnetite and between 45–250 µm, and between 4–15 µm, respectively, for the composition with 10%. Both of the compositions presented the typical topographic features of the nucleation mechanism and growth, with large perceptible increases (Figure 6a,d). The surface of the pore walls contained hill-style structures, with small peaks that gave the walls an appearance of micro-nano ruggedness (marked with green circles in Figure 6a,d) [33]. As the process of in-vitro degradation advanced, the pores both shrank and lengthened (Figure 6b,e); both of the compositions presented greater microporosity. In the PCL-5%nFe_3_O_4_ composition, the gradual appearance of micropores can start to be appreciated in a generalized way on the walls of the scaffold skeletons in week eight of degradation (Figure 6b), while in the composition with 10% nFe_3_O_4_, they were appreciable from week 16 onwards (Figure 6e). The pore walls, rather than not clean and smooth, were rugged, characteristic of the liquid-liquid separation through the nucleation mechanism and growth.

The magnetite particles were visible at higher magnifications after the degradation process at week 25, as can be clearly seen in Figure 6c (blue circles) for the composition with fewer particles.

While observing Figure 6c,e, it may be seen that there were more broken fibers and slightly deteriorated pores in week 25 of degradation: no degradation products were observed. This deterioration process provided the clearest morphological evidence of scaffold degradation, although the structural integrity of the scaffolds was maintained and no collapse of the structure occurred.

### 3.5. Water Absorption

The measurement of water absorption assisted the evaluation of the porous matrices of the PCL-nFe_3_O_4_ system. Other authors have previously observed a marked decrease in water absorption, which was coincident with a reduction in the mass of the sample and an increase in its dimensions [34,35]. In our study, no swelling of the scaffolds or any dramatic reduction in mass for any composition was observed. However, there were increases in water absorption that may be attributed to the absorption mechanism of PBS. PCL is a highly hydrophobic material and the absorption of water both in the scaffold pores and in the polymeric network was slow. During the first days in PBS, the pores of the scaffolds were filled with both air bubbles and water. The presence of those air bubbles persisted inside the internal scaffold pores, which blocled the penetration of water. As the water content never achieved equilibrium, it varied over time. Therefore, the variation in water absorption without the corresponding changes in the mass, the morphology, and/or the thermal properties in no way suggested the degradation of the porous matrices [33,34,35,36].

Moreover, the clearly alkaline property of the magnetite nanoparticles has to be considered, which can act as a physical barrier that blocks the entry of water into the scaffold, diminishing the speed of degradation, which can neutralize the degradation products, reducing the autocatalytic degradation of the polymer [36,37,38].

**Figure 7:**
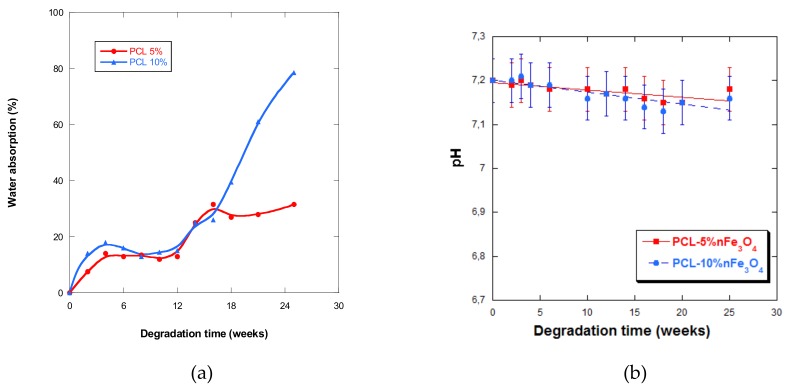
(**a**) Water absorption by PCL-5%nFe_3_O_4_ and PCL-10%nFe_3_O_4_ matrices vs. degradation time; (**b**) pH of the PBS solution vs. degradation time.

The curves in Figure 7a show that the scaffolds never absorbed large quantities of PBS during the first five weeks of incubation, after which water absorption slightly fluctuated up until week 12. As from week 5, it drastically increased up until week 25, especially for the composition with 10% nanoparticles. This behavior was very different from the one presented by the PCL-nHAFe system [22], which underwent rapid and greater absorption from week 5.

It was also observed that the higher the content of magnetite nanoparticles in the samples that were subjected to degradation, the higher the quantities of water that they were cable of absorbing, up until week 25, leading to a quicker degradation rate, a behavior that has also been observed by other authors [39]. However, absorption diminished in samples after two years of in-vitro degradation when the quantity of nanoparticles increased.

### 3.6. pH

The pH of the aqueous medium that varied with degradation time is a measure that indicates the liberation of acidic products, which reflects the advance of the degradation process. In all of the samples under study, a very small but sustained reduction in the pH (Figure 7b) was observed, except at week 4 of degradation in both scaffolds, when an increase in pH was observed. The fall in pH was minimal, due to the basic character of the nanoparticles or because the degradation products were themselves minimal.

### 3.7. Mass and Weight Loss

The in-vitro degradation process implies an alteration in the structure of matrices, which, in turn, means the degradation of the polymeric chain and a decrease in the molecular weight.

Over the first 25 weeks, no mass variation was obtained in the scaffolds with the lowest content of nanoparticles, obtaining a minimal loss of 2% in week 104 of degradation. Besides, the composition with 10% nFe_3_O_4_ underwent a slight loss of mass, less than 8%, as from week 14, with slight fluctuations always below that value throughout the degradation period.

Table 3 shows the values of Mw (average molecular weight by weight), Mn (average molecular weight by number), and I (polydispersity), which are directly related with the polymer chains and the dispersity of their molecular weights. The introduction of Fe_3_O_4_ nanoparticles evidently delayed the start of the degradation process, due to the slight variation in both the molecular mass and in the molecular weight at week 25 of degradation. As from week 25 of degradation, we can see that the Mw and the Mn of both samples diminished and the I of 1.459 increased to 1.617, due to the cleavage of the polymer chains that resulted from hydrolysis. In addition, the samples with fewer nanoparticles had a slightly higher I in week 104. The concentration of nFe_3_O_4_ had no great effect on the speed of sample degradation of the samples when they underwent a similar loss in molecular weight (Mw).

### 3.8. Cytotoxicity

**Figure 8:**
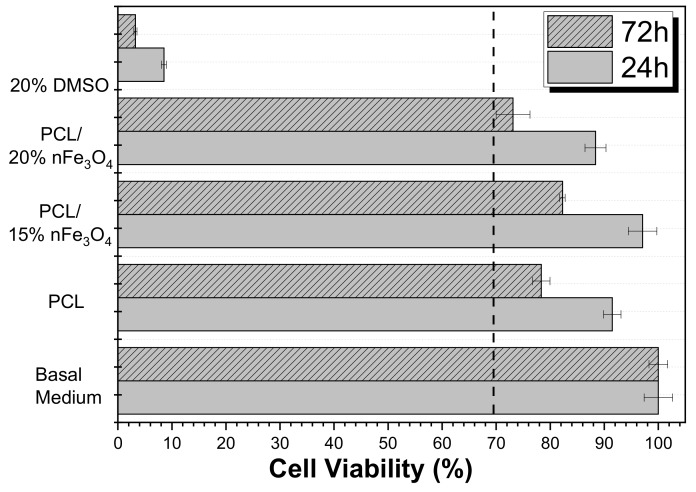
Cell Viability of MC3T3-E1 pre-osteoblast cell line in contact with the conditioned medium exposed with different samples after 24 and 72 h.

A cytotoxicity study of the materials that have been developed is essential for assessing their applicability for biomedical and tissue engineering applications. Thus, the effect of the polymer extract medium on the metabolic activity of MC3T3-E1 pre-osteoblast cells was evaluated while using MTT after 24 and 72 h (Figure 8).

The use of poly(ε-caprolactone) (PCL) as a scaffold material is not restricted to one type of tissue due to its adaptability. It is a biocompatible polyester that has been widely used in tissue engineering based on its availability, its relative inexpensiveness, and its suitability for specific modifications [35,37]. Figure 8 shows that neat PCL is non-cytotoxic up to 72 h (cell viability reduction is less than 30%), as reported in the literature. According to the ISO standard 10993-5, the samples are only considered to be cytotoxic when the cell viability reduction is more than 30%. Although the Fe_3_O_4_ nanoparticles are cytotoxic at certain concentrations [38], the composites PCL/Fe_3_O_4_ are non-cytotoxic. Nevertheless, it was noted that the scaffolds with 20% Fe_3_O_4_ were at the limit of their cytotoxicity. These results showed that the nanoparticles were properly encapsulated within the biocompatible polymer, which agrees with previous studies on related polymers [39,40,41].

Therefore these materials are reliable alternatives for bone tissue engineering and can also be explored in dynamic cell culture through suitable magnetic stimulation.

## 4. Conclusions

The Thermally Induced Phase Separation (TIPS) method has been shown to be a promising and simple method for the manufacture of porous magnetic matrices of polycaprolactone with magnetite nanoparticles. The addition of nanoparticles facilitated the absorption of PBS, with no greater increase in the degradation speed of the matrices, as demonstrated by the results of Mw, Mn, and I. The study of matrix morphology through SEM appeared to not point to a degradation process over the period of this study, with the exception of pore size reduction. The size of the nanoparticles determined the ferromagnetic behavior of the scaffolds, which were non-cytotoxic for the MC3T3-E1 pre-osteoblast cells.

## Figures and Tables

**Table 1:** Magnetization at 1.5 Tesla relative to the nominal content of nFe_3_O_4_, coercive field, and actual content of nFe_3_O_4_ recalculated from the measurements. Estimated errors are displayed in brackets and refer to the variation of the last significative figure.

% nFe_3_O_4_ Nominal	Magnetization at 1.5T (Am^2^/kg _nFe3O4_)	µ_0_ H_c_ (mT)	%nFe_3_O_4_ Recalculated
100 *	82 (2)	11.9 (2)	-
5	78 (2)	12.0 (2)	4.8 (1)
10	81 (2)	11.9 (2)	9.9 (2)
15	75 (1)	11.1 (2)	13.8 (3)
20	69 (1)	11.4 (2)	16.8 (4)

* Pure nFe_3_O_4_ serving as a reference.

**Table 2:** Parameters obtained by DSC on the PCL-nFe_3_O_4_ scaffolds: Tm = melting point (°C), ΔH_m_ = melting enthalpy (kJ/kg), Tc = crystallization temperature (°C), ΔH_c_ = crystallization enthalpy (kJ/kg), T_g_ = glass transition temperature (°C), Xc = crystalline fraction (%), calculated as Xc% = [(ΔH_m1_ − ΔH_c_)/ΔH_mo_] × 100% with ΔH_mo_ = 139.5 J·g^−1^, CF% = (ΔH_c_/ΔH_m1_) × 100%.

Sample PCL dt (weeks)	1st Run		2nd Run		3rd Run		T_g_ (°C)	X_c_%	CF%
	T_m1_ (°C)	ΔH_m1_ (J·g^−1^)	T_c_ (°C)	ΔH_c_ (J·g^−1^)	T_m2_ (°C)	ΔH_m2_ (J·g^−1^)			
0%nFe_3_O_4_	60	107.4	26	64.7	57	83.2	−59	77	60.2
5%nFe_3_O_4_ 0dt	60	94.6	30	60.2	58	74.1	−59	68	64
4dt	63	93.9	31	59.2	57	65.6	−58.5	67	63
8dt	64	88.7	30.5	58.6	58	65.4	−60	64	66
16dt	63	90	30	58.1	58	65.4	−63	65	65
20dt	67	80.5	31.5	53	56.5	72.9	−63	58	65
25dt	67	77.7	31	52.4	56	68.5	−63	56	67
104dt	65	90.2	31	57.4	58	59.7	−64	65	64
10%nFe_3_O_4_ 0dt	59	92.2	31	59.9	56	73.6	−59	66	65
4dt	63	67	31	47.2	57.5	49	−58	48	70
8dt	64	91	31	59.3	58	66.9	−61	65	65
16dt	63	80.2	31	49.7	58	64.4	−58	57	62
20dt	66	77.5	30.5	49.4	55.5	57.6	−63	56	62
25dt	67	76.9	32	48	56	54.7	−63	55	62
104dt	65	94.3	31	55.4	57	62.2	−63	68	59
15%nFe_3_O_4_ 0dt	59	83.1	30	52	59	69.7	−58	60	63
20%nFe_3_O_4_ 0dt	58	81.3	30	51	58	65.9	−58	58	63

**Table 3:** Molecular weight: weight average (Mw), number average (Mn), and polydispersity index I of the PCL-nFe_3_O_4_ system.

Sample	Degradation Time (Weeks)	Mw	Mn	I
PCL	0	134418	92103	1.459
PCL-5%nFe_3_O_4_	25	143410	97073	1.477
	104	99888	61754	1.617
PCL-10%nFe_3_O_4_	25	139433	90264	1.545
	104	86212	54070	1.594
PCL-15%nFe_3_O_4_	0	134441	95866	1.402
